# Supplementary material for: Postindustrial Landscapes Are Neglected Localities That May Play an Important Role in the Urban Ecology of Ticks and Tick-Borne Diseases—A Pilot Study
Source: Pathogens. 2023 Apr 27;12(5):648. doi: 10.3390/pathogens12050648 (PMC10220862; doi:10.3390/pathogens12050648)
Supplement: Supplementary file 1 [file pathogens-12-00648-s001.zip › Figure S1--S11.jpeg.pdf]

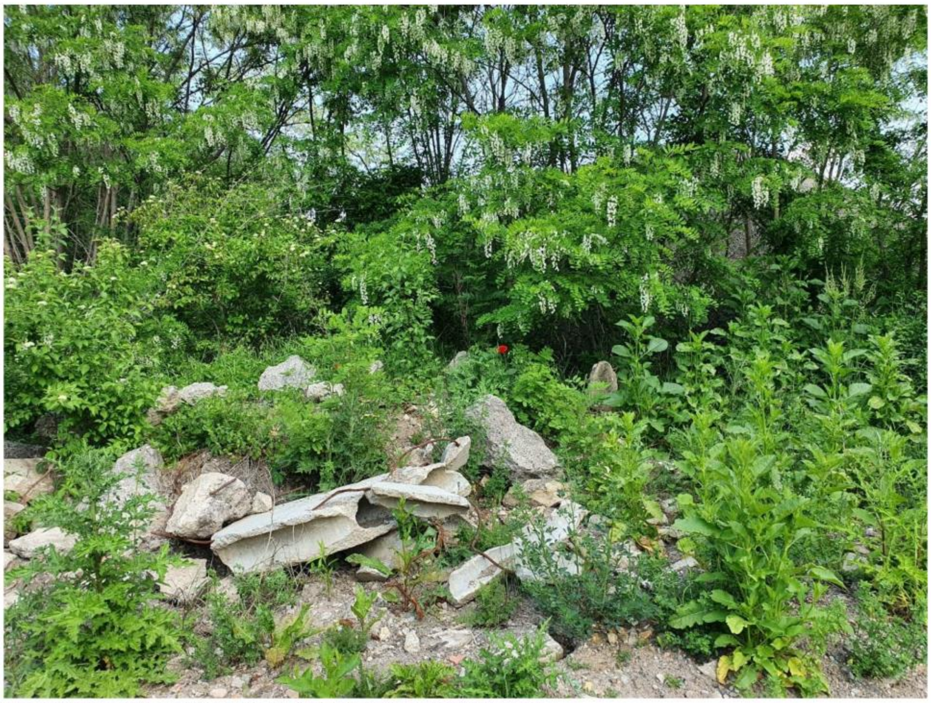

Figure S1. Construction waste material of the deposit. Dvořáková 2021.

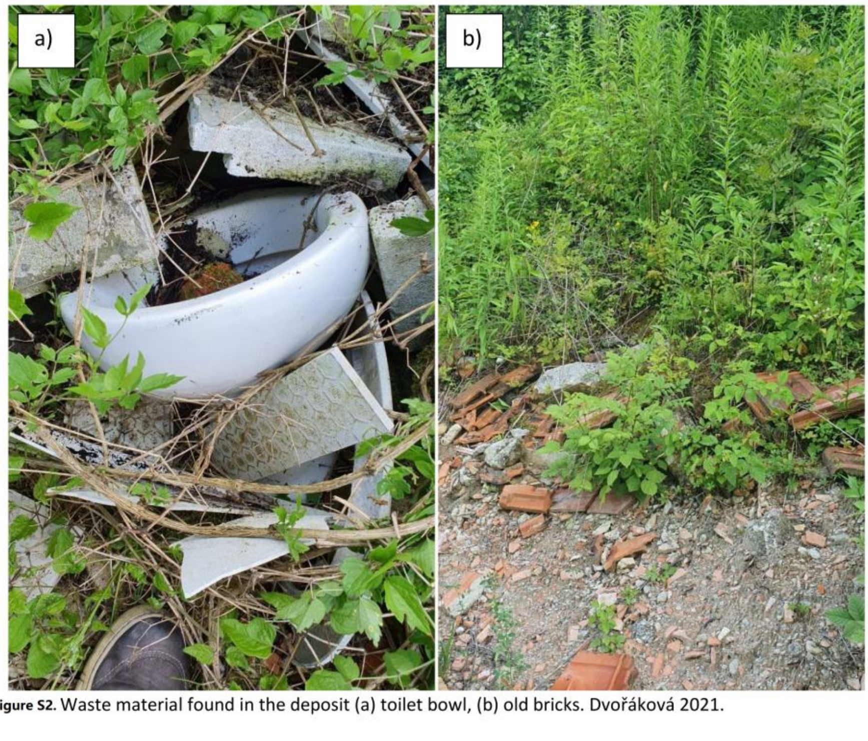

Figure S2. Waste material found in the deposit (a) toilet bowl, (b) old bricks. Dvořáková 2021.

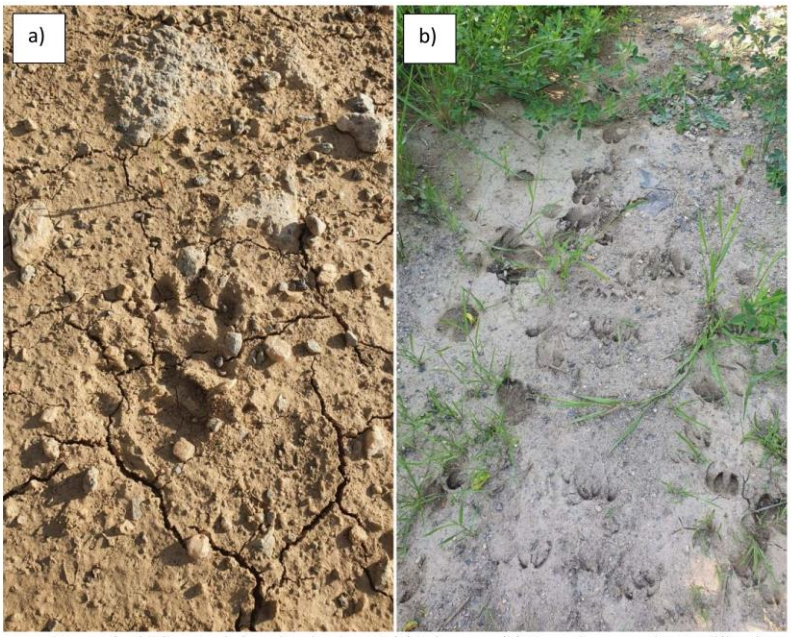

Figure S3. Traces of wildlife animals found in the deposit (a) *Felis catus,* (b) *Capreolus capreolus*. Dvořáková 2021

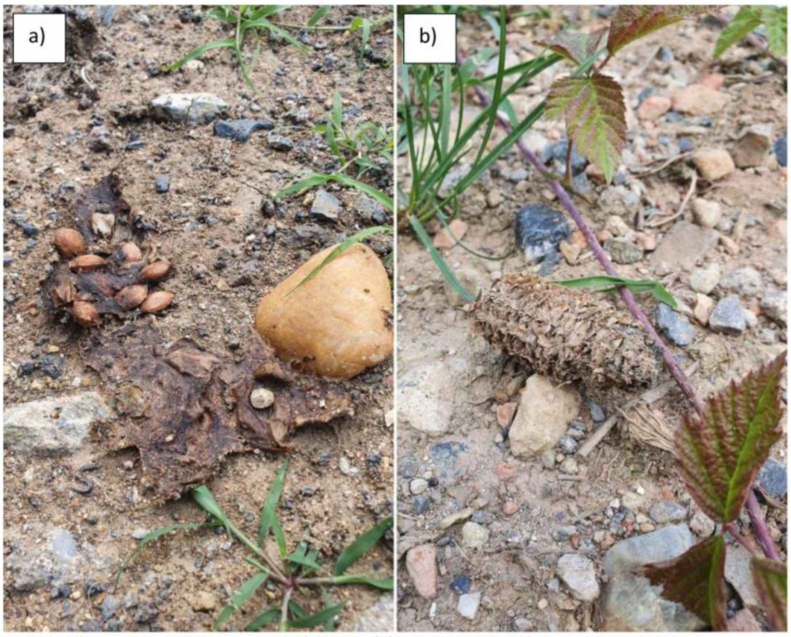

Figure S4. Habitat signs found in the deposit (a) feces, (b) gnawed ear of *Zea mays*. Dvořáková 2021.

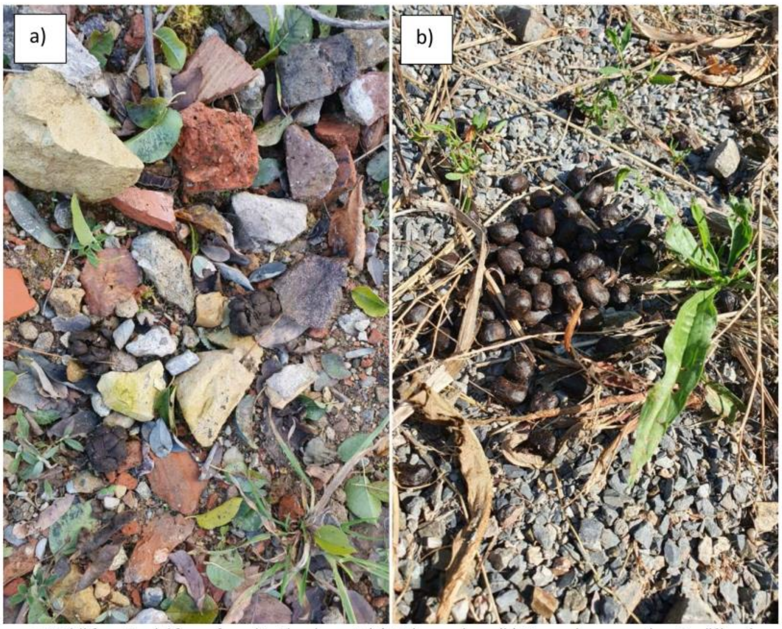

Figure S5. Wildlife animals' feces found in the deposit (a) *Vulpes vulpes*, (b) *Capreolus capreolus*. Dvořáková 2021

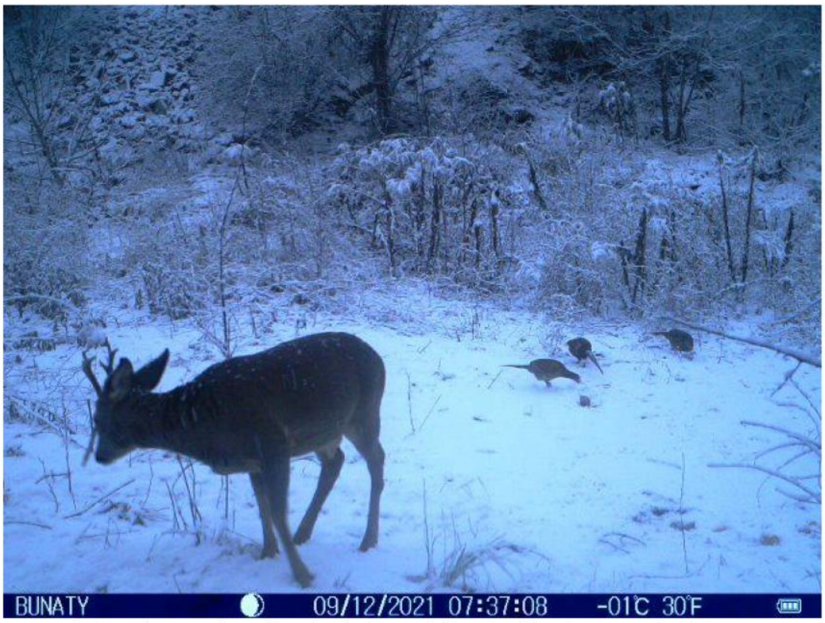

Figure S6. Male *Capreolus capreolus* and females *Phasianus colchicus*. Czech hunting community 2021.

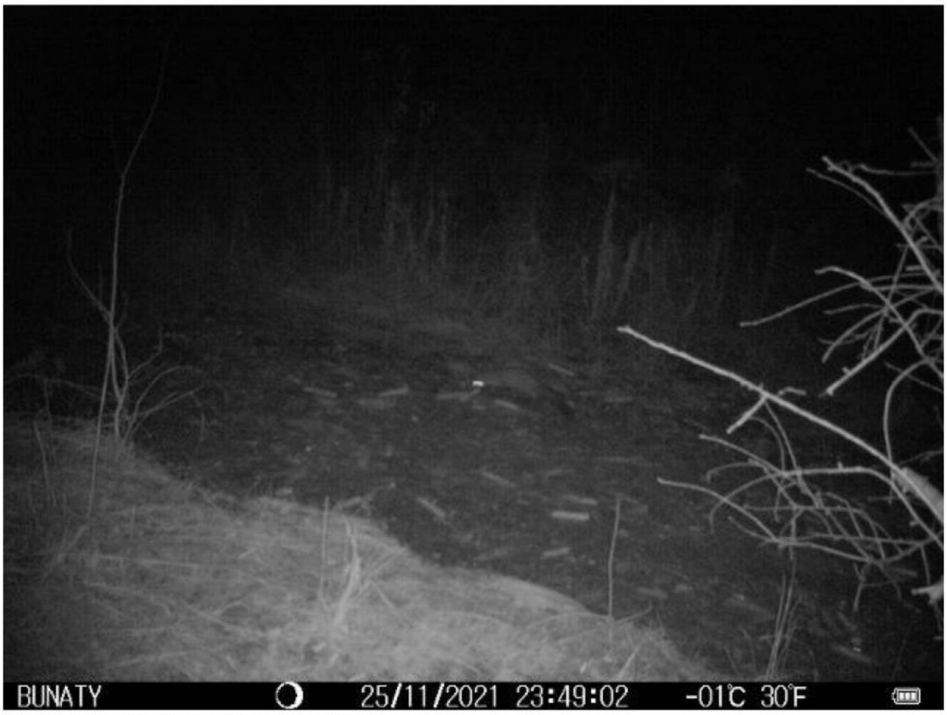

Figure S7. *Martes martes*. Czech hunting community 2021.

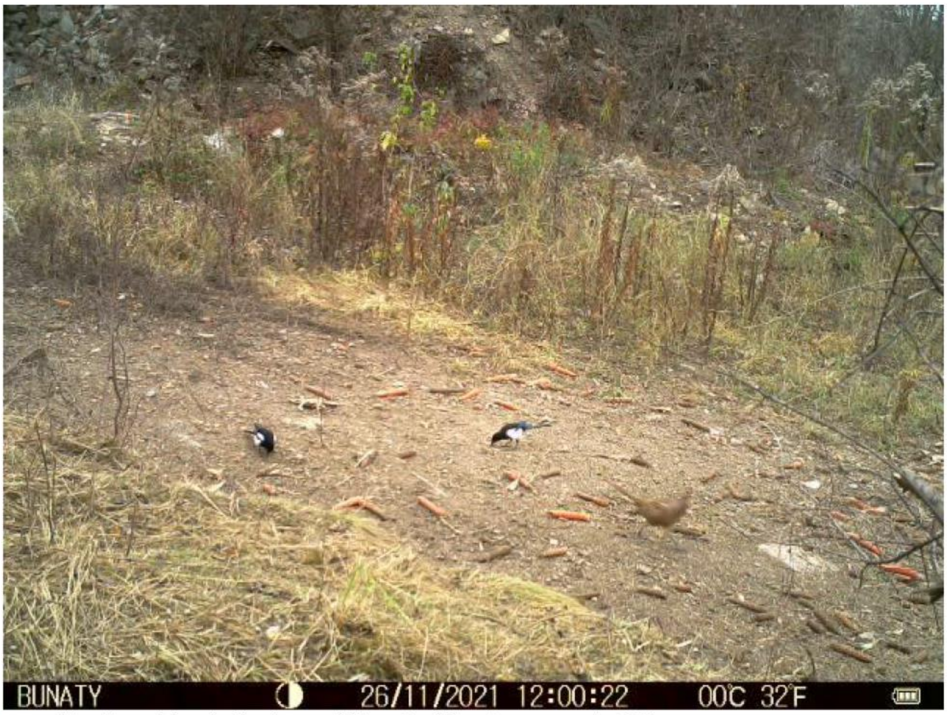

Figure S8. *Pica pica* and female *Phasianus colchicus*. Czech hunting community 2021.

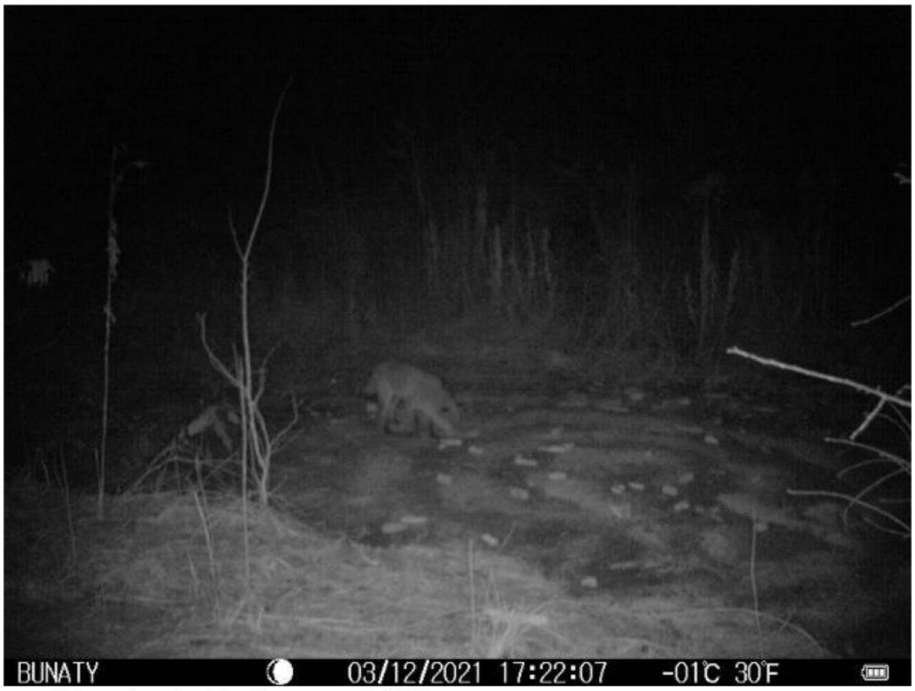

Figure S9. *Vulpes vulpes*. Czech hunting community 2021.

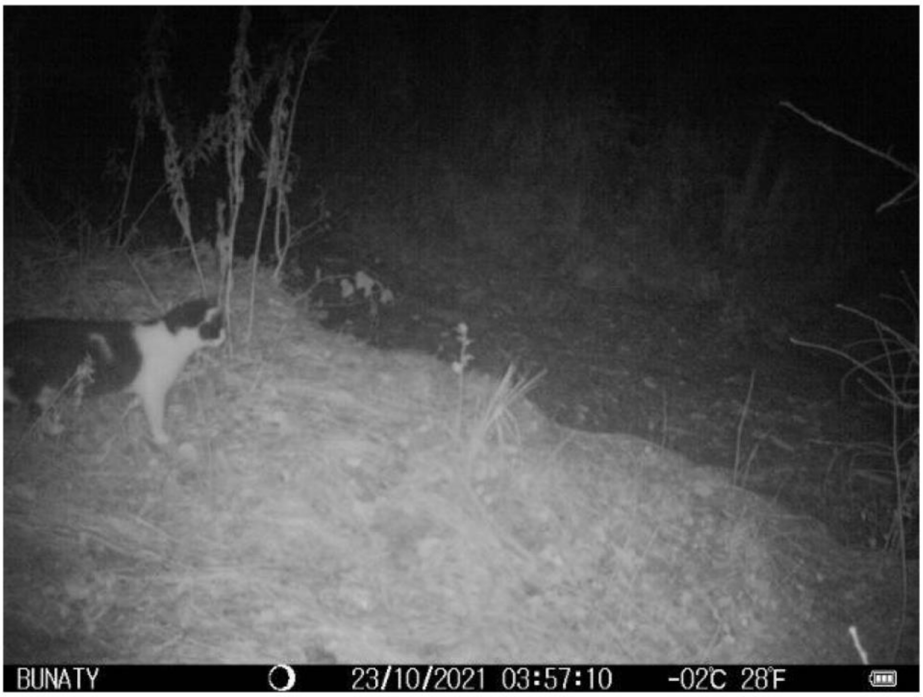

Figure S10. *Felis catus*. Czech hunting community 2021.

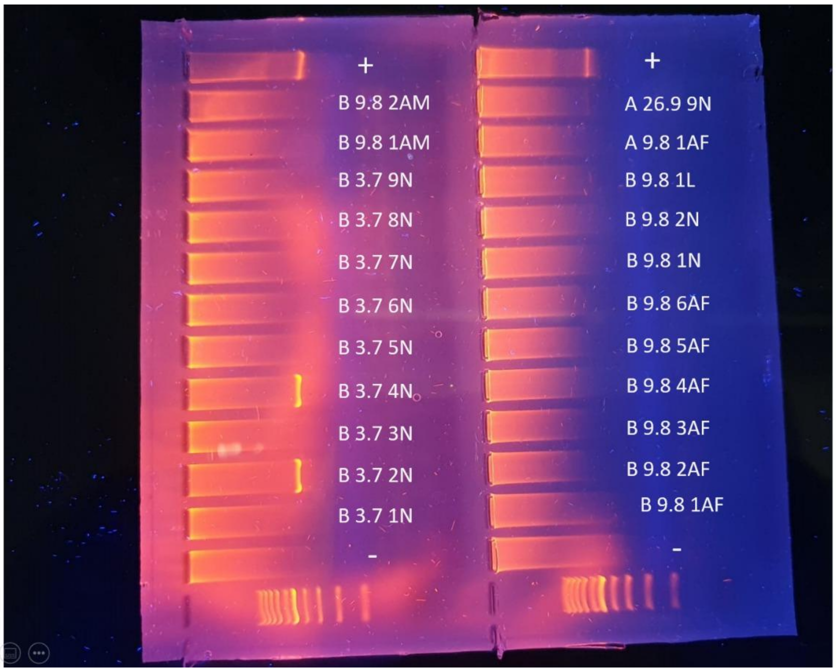

Figure S11. Example of an electrophoresis gel with a positive result and adequate controls.
